# Protein Crosslinking by Transglutaminase Controls Cuticle Morphogenesis in *Drosophila*

**DOI:** 10.1371/journal.pone.0013477

**Published:** 2010-10-18

**Authors:** Toshio Shibata, Shigeru Ariki, Naoaki Shinzawa, Ryuta Miyaji, Haruka Suyama, Miyuki Sako, Nobuyuki Inomata, Takumi Koshiba, Hirotaka Kanuka, Shun-ichiro Kawabata

**Affiliations:** 1 Graduate School of Systems Life Sciences, Kyushu University, Fukuoka, Japan; 2 Department of Biology, Faculty of Sciences, Kyushu University, Fukuoka, Japan; 3 National Research Center for Protozoan Diseases, Obihiro University of Agriculture and Veterinary Medicine, Obihiro, Hokkaido, Japan; Indiana University, United States of America

## Abstract

Transglutaminase (TG) plays important and diverse roles in mammals, such as blood coagulation and formation of the skin barrier, by catalyzing protein crosslinking. In invertebrates, TG is known to be involved in immobilization of invading pathogens at sites of injury. Here we demonstrate that *Drosophila* TG is an important enzyme for cuticle morphogenesis. Although TG activity was undetectable before the second instar larval stage, it dramatically increased in the third instar larval stage. RNA interference (RNAi) of the *TG* gene caused a pupal semi-lethal phenotype and abnormal morphology. Furthermore, *TG*-RNAi flies showed a significantly shorter life span than their counterparts, and approximately 90% of flies died within 30 days after eclosion. Stage-specific *TG*-RNAi before the third instar larval stage resulted in cuticle abnormality, but the *TG*-RNAi after the late pupal stage did not, indicating that TG plays a key role at or before the early pupal stage. Immediately following eclosion, acid-extractable protein from wild-type wings was nearly all converted to non-extractable protein due to wing maturation, whereas several proteins remained acid-extractable in the mature wings of *TG*-RNAi flies. We identified four proteins—two cuticular chitin-binding proteins, larval serum protein 2, and a putative C-type lectin—as TG substrates. RNAi of their corresponding genes caused a lethal phenotype or cuticle abnormality. Our results indicate that TG-dependent protein crosslinking in *Drosophila* plays a key role in cuticle morphogenesis and sclerotization.

## Introduction

In mammals, TG fulfills a variety of essential functions by catalyzing isopeptide bond formation between Lys and Gln residues to form ε-(γ-glutamyl) lysine bonds between appropriate substrates in a Ca^2+^-dependent manner [1]–[3]. For example, plasma TG (factor XIII) stabilizes noncovalently associated fibrin polymers through covalent crosslinking of substituent fibrin monomers [4], and TG-1 (keratinocyte TG) crosslinks several proteins to form a thick layer of insoluble proteins, resulting in the formation of a cornified cell envelope [2]. In invertebrates, such as the crayfish *Pacifastacus leniusculus* and *Drosophila*, hemolymph coagulation depends on TG-mediated crosslinking of specific clotting proteins [5]–[9]. In the horseshoe crab *Tachypleus tridentatus*, a proteolytic coagulation cascade leads to the conversion of coagulogen into insoluble coagulin polymers, which are in turn stabilized by TG-mediated crosslinking with TG substrates including proxin and stablin, resulting in immobilization of invading pathogens at sites of injury [10]–[13]. On the other hand, in the nematode parasite *Onchocerca volvulus*, TG-catalyzed crosslinking is important for the molting of third-stage larvae [14]. TGase activity is also important in hemocyte homeostasis in the hematopoietic tissue of *P. leniusculus* [15]. Recently, Wang *et al*. provided proof for an immune function for *Drosophila* TG: *Drosophila* larvae with reduced TG levels exhibit increased mortality after septic injury and are more susceptible to a natural infection involving entomopathogenic nematodes and their symbiotic bacteria [16].

Arthropod cuticles function principally as an exoskeletal covering of the total body surface and are highly organized structures produced by extracellular secretion from the epidermis. Cuticles consist of chitin filaments, proteins, lipids, and inorganic substances, which are modified by sclerotic processes such as the oxidative incorporation of *o*-diphenols into the cuticle matrix [17]–[20]. We have observed TG-dependent crosslinking of cuticle proteins in the horseshoe crab and characterized one of the cuticle proteins specifically expressed in epidermis, which we designated caraxin [21], [22]. Crosslinked caraxin forms an elaborate mesh of honeycomb structures, suggesting that the mesh plays a role in promoting wound healing and sclerotization at sites of injury. Thus, as in the case of mammalian skin, TG-dependent protein crosslinking may be involved in the initial stage of host defense in the sub-cuticular epidermis of arthropods. The TG family comprises eight members in mammals, with each member performing diverse physiological functions [3]. In contrast, genome annotations in *Drosophila* have identified only a single *TG* gene that is predicted to encode a protein of 87 kDa (CG7356).

Here we characterized *Drosophila* TG biochemically and genetically, and demonstrated that the invertebrate TG is involved in cuticle morphogenesis and sclerotization *in vivo*. The epidermis-barrier function against invading pathogens and the wound-repair pathway appear to be evolutionarily well conserved between mammals and *Drosophila*, indicating that *Drosophila* would likely serve as a sophisticated model system for elucidation of the molecular mechanisms underlying mammalian skin disorders.

## Results

### Stage-specific expression pattern of TG and effects of wounding on *TG* expression

**Figure 1:**
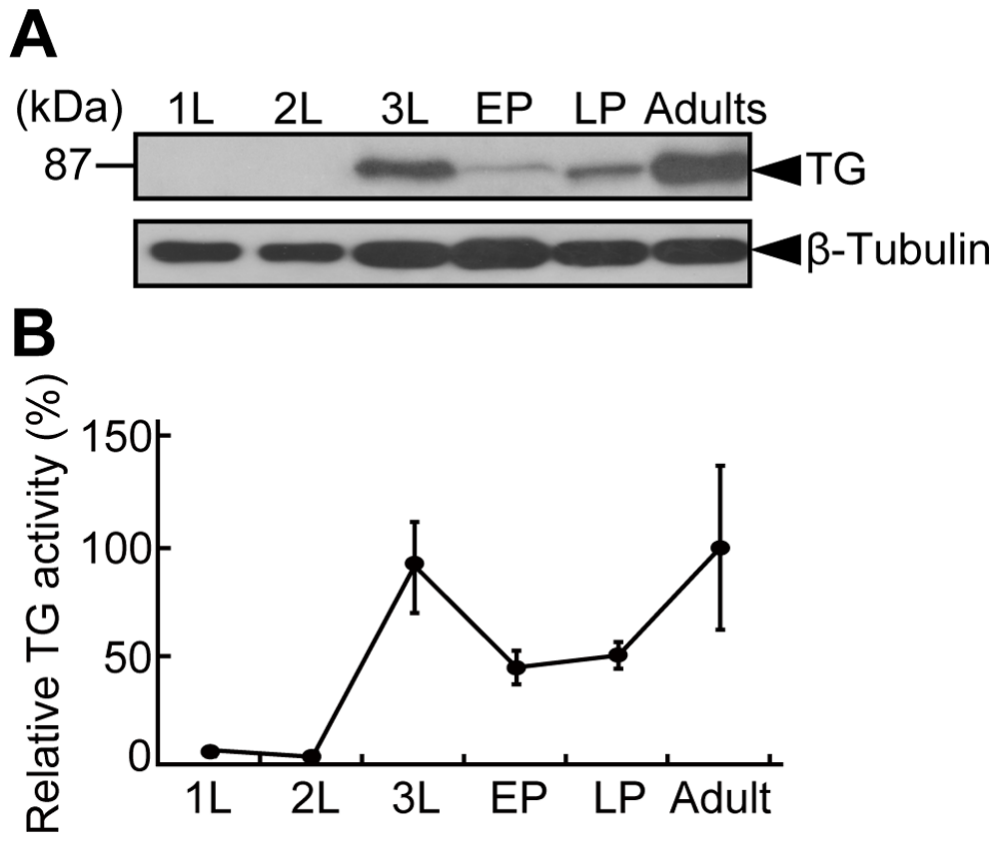
Stage-specific expression of *TG*. (A) The wild-type flies were collected at indicated developmental stages and homogenized. TG antigens were detected by Western blotting (upper panel). β-tubulin was detected by Western blotting as a control (lower panel) with a mouse anti-tubulin antibody. (B) TG activity was assayed by the incorporation of Bi-PA into *N, N'*-dimethylcasein. The means ± S. D. of three independent experiments were plotted.

**Figure 2:**
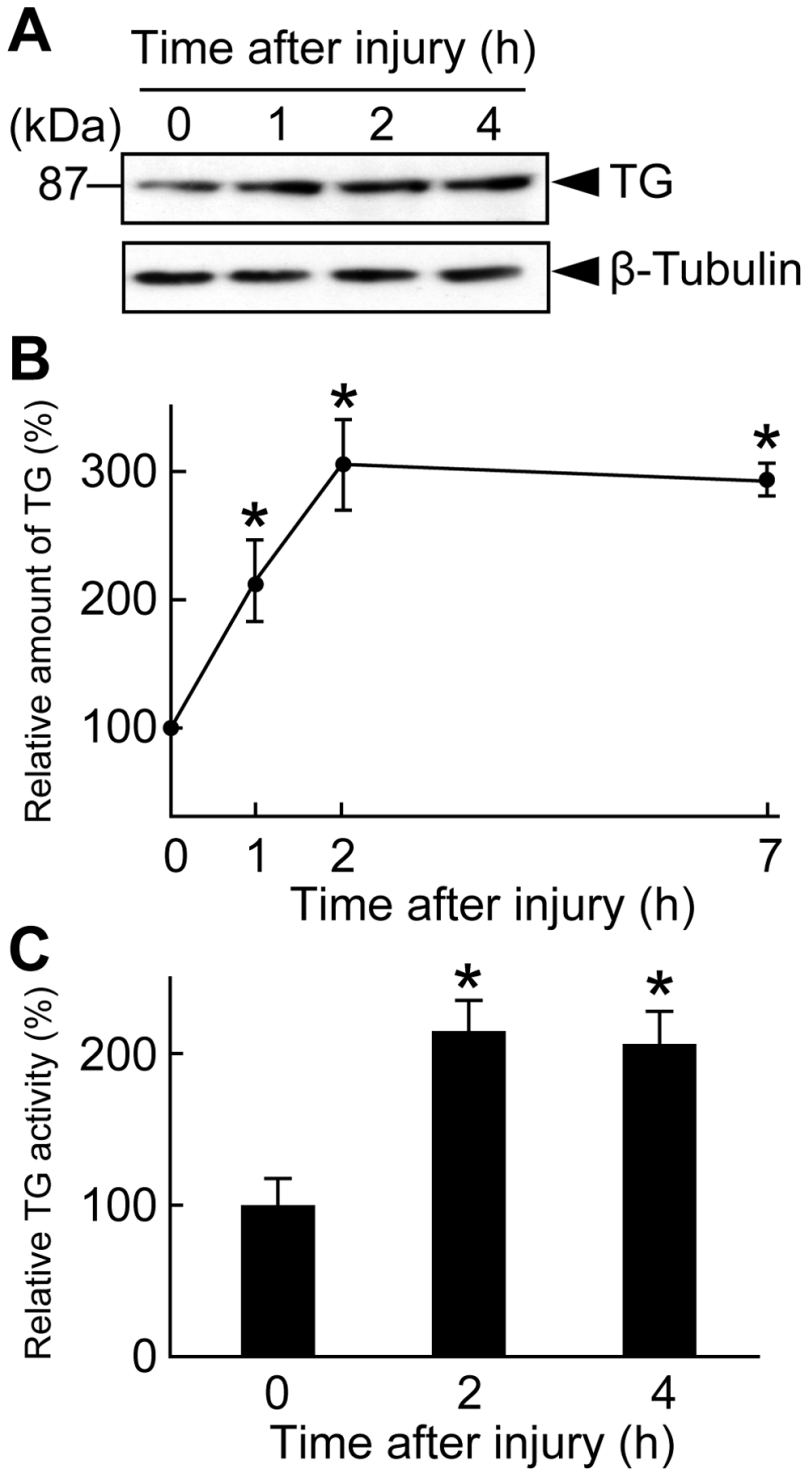
The effect of wounding on *TG* expression. Wild-type flies were injured using a steel pin. Flies were collected at the indicated times and homogenized. (A) TG antigens at the indicated times were detected by Western blotting (upper panel). β-tubulin was detected by Western blotting as control (lower panel). (B) The amount of TG after the wounding was determined by enzyme-linked immunosorbent assay. The means ± S. D. of three independent experiments were plotted. A significant difference (versus 0 h) is indicated by asterisk (*P*<0.05 after Bonferroni correction). (C) TG activities were measured by the monodansylcadaverine incorporation at 1 and 4 h after wounding. The means ± S. D. of three independent experiments were plotted. A significant difference (versus 0 h) is indicated by asterisk *(P*<0.05).

The amount of TG antigen and the TG enzyme activity in the extract of whole body were evaluated at different developmental stages. TG activity was significantly different between developmental stages (*F*_5, 12_ = 15.178, *P*<0.0001 by the ANOVA analysis) (Figure 1B). Although neither TG antigen nor TG activity were detectable at the first instar larva (1L) or second instar larva (2L) stage, both were clearly detected after the third instar larva (3L) stage (Figures 1A and 1B). The differences in TG activity between 1L and 3L or 2L and 3L were significant at 5% level (the Bonferroni correction for multiple comparisons). TG activity in adult flies was equivalent to that of 3L (Figure 1B). To evaluate whether *TG* expression is induced in response to stimulation by wounding, adult flies were injured by a steel pin at the abdominal sternite. TG antigen increased at 1 h after injury (Figure 2A). Consistent with this observation, an approximately threefold increase in TG antigen was observed by ELISA at 2 h after injury, with the antigen level persisting for at least 4 days (Figure 2B: *F*_3, 8_ = 24.182, *P* = 0.0002). The differences in TG antigen between 0 h and 1 h, 0 h and 2 h, or 0 h and 7 h were significant at 5% level. Moreover, an increase in TG activity in response to wounding was observed by TG enzymatic activity assay, further supporting the hypothesis that TG is involved in wound repair in *Drosophila* (Figure 2C: *F*_2, 6_ = 8.8906, *P* = 0.01606). The differences in TG activity between 0 h and 2 h or 0 h and 4 h were significant at 5% level.

### Phenotypes of *TG*-RNAi flies

**Figure 3:**
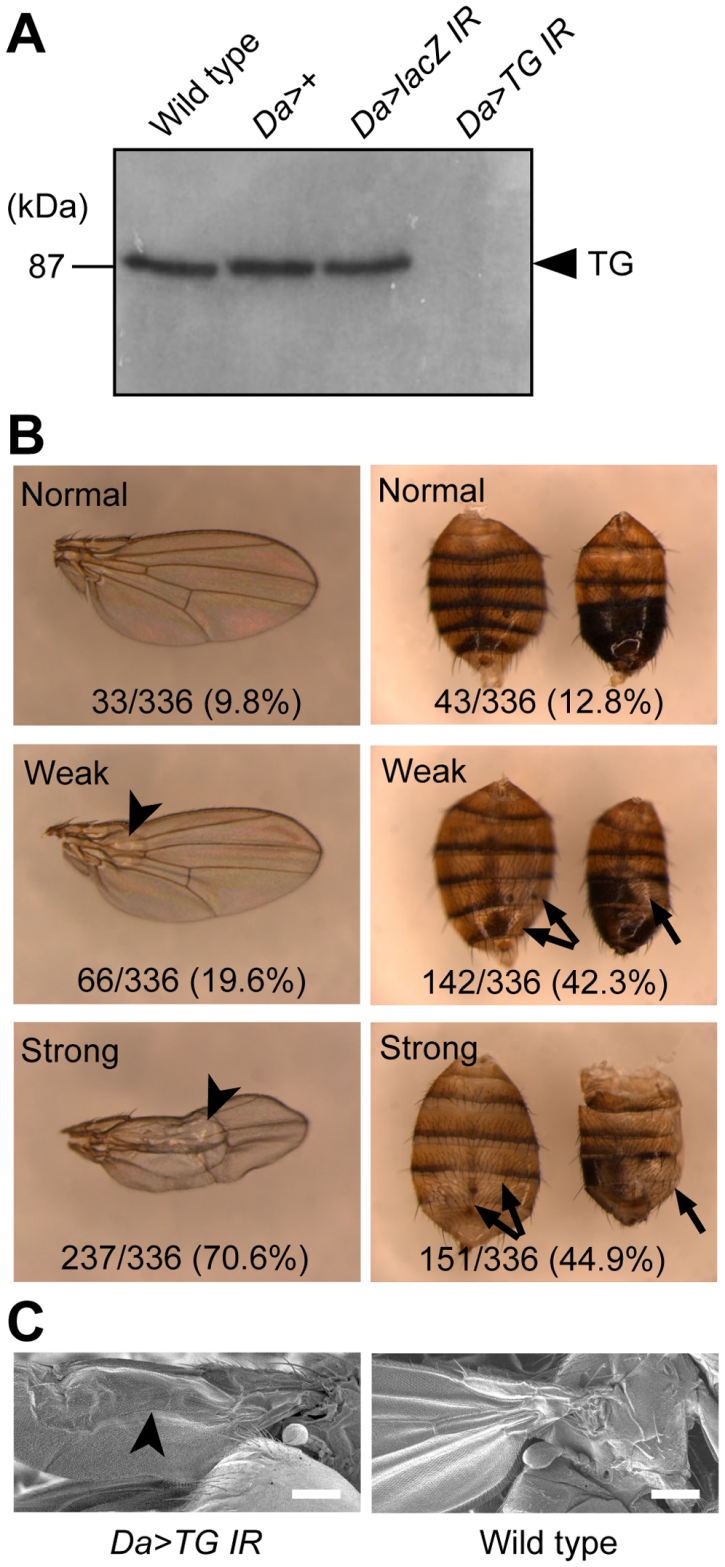
Phenotypes of *TG*-RNAi flies. (A) TG antigen from whole body extract of adult *TG*-RNAi flies was detected by Western blotting. *w^1118^*, *Da-GAL4>+* and *Da-GAL4>UAS-LacZ IR* were used as controls. *Da*, *Da-GAL4*. (B) Phenotypes of *TG*-RNAi flies for the wing (left panels) and abdominal cuticle (right panels) were classified into three grades depending on the extent of observed abnormality. The ratios of abnormal flies to total adult flies are indicated. Each fly was laid at 25°C. (C) Scanning electron microscopy of *TG*-RNAi fly. Scale bar = 200 µm.

**Figure 4:**
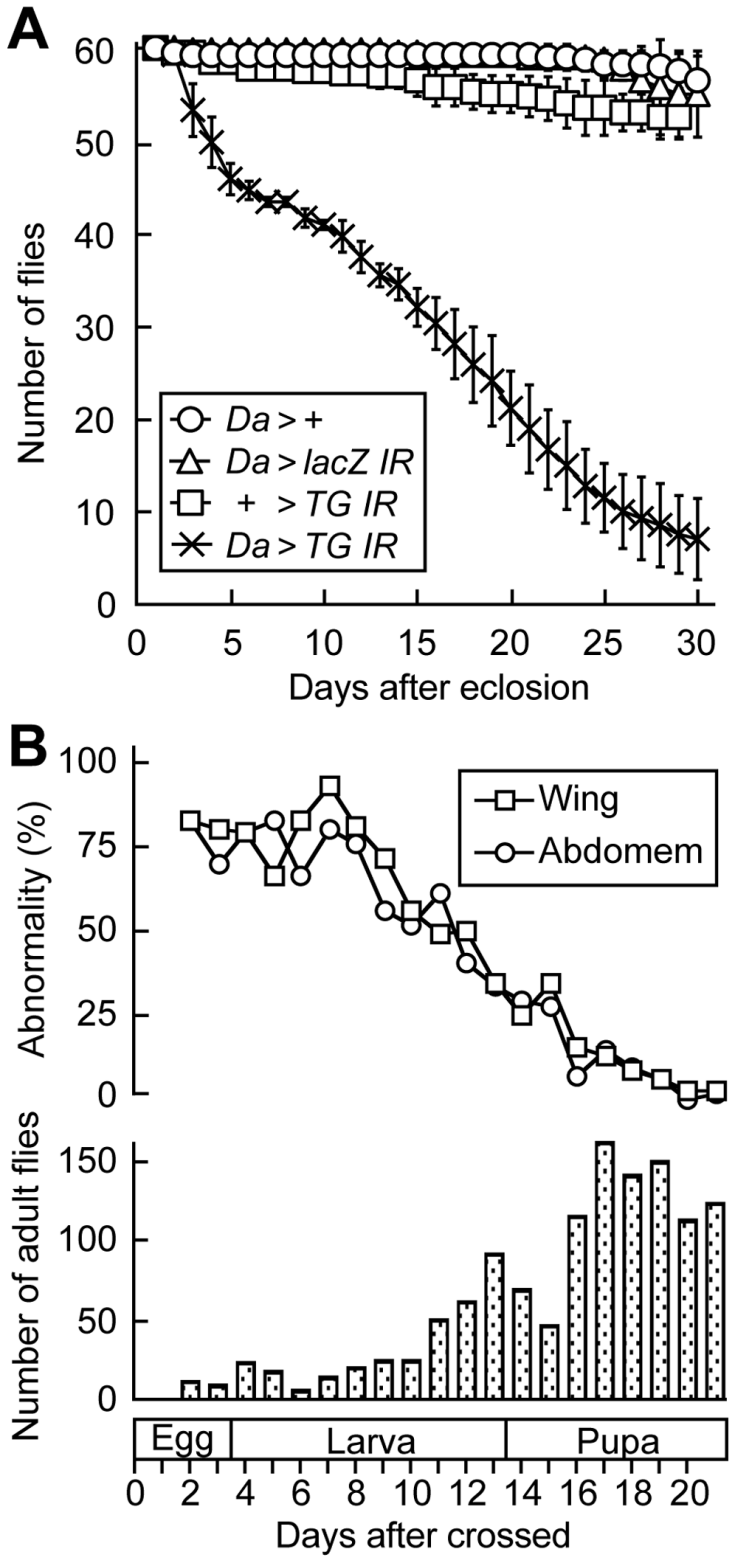
The life span of the *Da-GAL4>UAS-TG IR* flies. (A) The life span of the RNAi flies was compared with those of the control flies, *Da-GAL4>+*, *Da-GAL4>UAS-lacZ IR* and *+>UAS-TG IR*. Sixty adult flies were collected and maintained at 25°C. The number of surviving flies was recorded daily. The means ± S. D. of four independent experiments were plotted. *Da*, *Da-GAL4*. (B) Phenotypes of *Tub-GAL80^ts^; Da-GAL4>UAS-TG IR*. *Tub-GAL80^ts^; Da-GAL4* flies were crossed with the *UAS-TG IR* flies in 20 vials and maintained at 18°C. The suppression of *TG* by RNAi was triggered by increasing the temperature to 29°C. The ratios flies with abnormal wings (square) and abnormal abdominal cuticles (circle) to total adult flies are indicated (upper panel). The number of adult flies born from each vial is indicated (lower panel).

We next characterized the phenotypes of *TG*-RNAi flies using ubiquitously expressed driver (*Da-GAL4>UAS-TG IR*). No TG antigen was detected in the extract from whole body of *TG*-RNAi flies by western blotting (Figure 3A, *Da>TG IR*). *TG*-RNAi revealed a pupal semi-lethal phenotype, with an eclosion rate for *TG*-RNAi flies that was about 20% that of controls expressing a *LacZ*-RNAi construct (*Da>lacZ IR*). After eclosion, about 90% of *TG*-RNAi flies exhibited abnormal cuticle morphologies of the wings and the abdominal tergite (Figure 3B). Wing formation is executed by well-defined stages; upon pupation the imaginal wing discs evaginate, and subsequent epithelial cell expansion without further cell proliferation causes the wings to become compactly folded within the confines of the pupal case and to spread within 1 h after eclosion [23], [24]. *TG*-RNAi flies, however, failed to expand their wings, causing the wings to blister (Figures 3B and 3C, arrowheads). In addition, several melanized segments on the abdominal tergite of adult flies failed to develop in *TG*-RNAi flies (Figure 3B, arrows). *TG*-RNAi flies showed a significantly shorter life span than did their wild-type counterparts, with approximately 90% of the flies dying within 30 days of eclosion (Figure 4A; χ^2^ = 11084.091, d.f. = 1. *P*<0.0001). In contrast, control flies, *Da-GAL4>+*, *Da-GAL4>UAS-LacZ IR*, and *+>UAS-TG IR*, developed normally into adults and did not display any cuticle abnormalities.

In order to investigate the role of TG at different developmental stages, we used a temperature-sensitive mutant (*Tub-GAL80^ts^*; *Da-GAL4*) in which the driver function of GAL4 is repressed by ubiquitously expressed GAL80 at 18°C and de-repressed at 29°C, thereby enabling selective temporal expression of the *UAS-TG IR.* To obtain the temperature-sensitive flies, *Tub-GAL80^ts^; Da-GAL4* flies were crossed with *UAS-TG IR* flies in 20 vials for 2 days at 18°C, and then, each vial containing eggs was transferred to 29°C at one-day interval. After 21 days of the crossing, the numbers of matured flies in the vials were counted. The stage-specific RNAi before 3L significantly increased the frequency of lethality, and about 75% of the adult flies had abnormalities in the formation of their wings and abdominal stripes (Figure 4B). In contrast, the RNAi after LP had no effect on cuticle abnormality (Figure 4B), indicating that the importance of TG in cuticle morphogenesis is particularly pronounced prior to the EP stage.

### Identification of TG substrates

**Figure 5:**
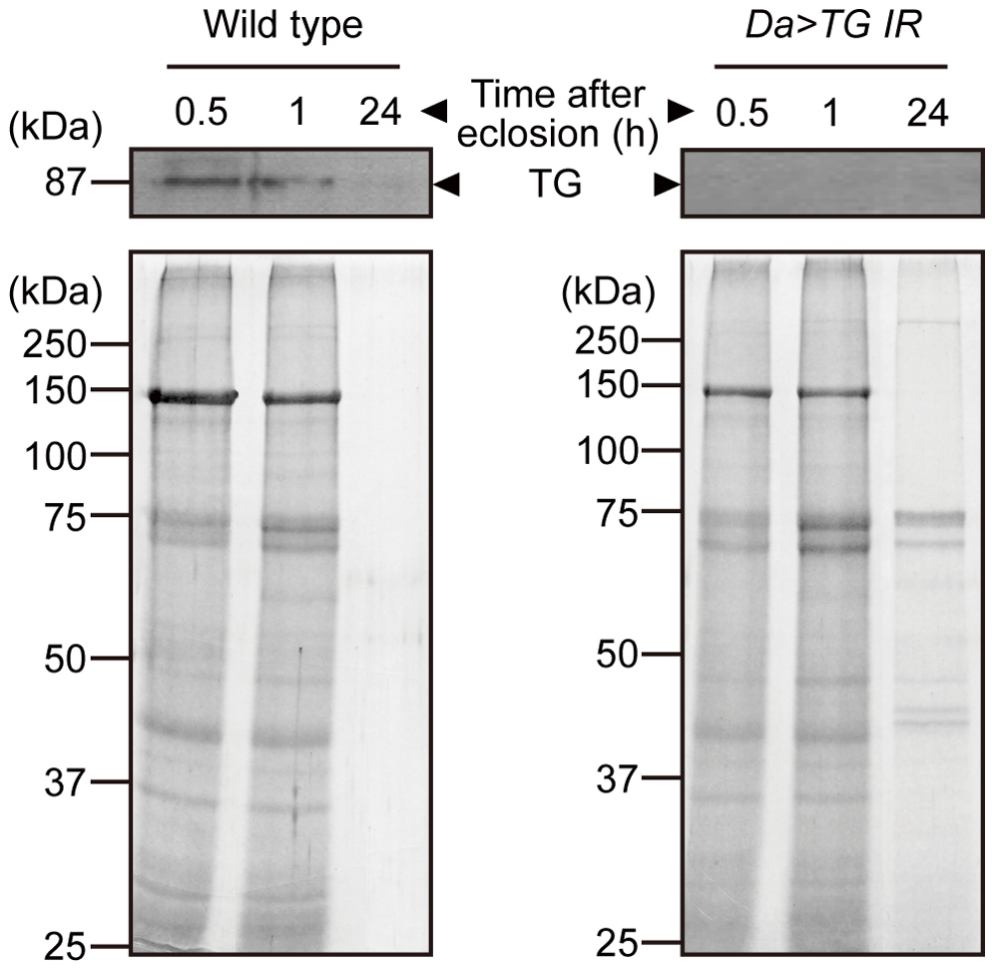
Identification of TG substrates associated with cuticle formation. The wings of wild-type and *Da-GAL4>UAS-TG IR* flies were collected at indicated times after eclosion. Wing proteins were extracted and subjected to SDS-PAGE. TG antigen was detected by Western blotting (upper panels). Loaded proteins were stained with Coomassie Brilliant Blue R-250 (lower panels). *Da, Da-GAL4.*

To identify TG substrates, cuticle proteins were extracted with 10% acetic acid from the wings of wild-type (*w^1118^*) and *Da-GAL4>UAS-TG IR* flies after eclosion. In wild-type flies, proteins that were acid-extractable immediately after eclosion (0.5 h) disappeared by 24 h post-eclosion, raising the possibility that during wing maturation these proteins are crosslinked to generate non-acid-extractable forms (Figure 5, Wild type). In contrast, several proteins were still extractable from the wings of *TG*-RNAi flies at 24 h post-eclosion, implying that they are candidates for TG substrates (Figure 5, *Da>TG IR*). These proteins were subjected to mass spectrometry, resulting in the identification of 12 proteins (Table 1).

**Table 1:** Acid-extractable wing proteins from *TG*-RNAi flies identified by mass spectrometry.

Nominal molecular weight	CG number	Protein name	MASCOT score	Sequence coverage (%)	Phenotype with *Da-GAL4*	Phenotype with *MS-GAL4*
374386	CG11064	RFABP	5117	54	Lethal	NP
124155	CG9299	Cpr76Bd	974	32	Faded melanization (See Fig. 6)	NP
83409	CG6806	LSP2	2248	60	Faded melanization (See Fig. 6)	NP
72964	CG6186	Transferrin 1	1826	82	Lethal	NP
50573	CG13214	Cpr47Ef	985	36	Lethal	NP
48802	CG1780	Imaginal disc growth factor 4	1036	70	NP	NP
29372	CG10287	Gasp	931	85	NP	NP
26900	CG3244	Clect27	461	48	Lethal	Anterior crossvein loss (See Fig. 6)
26735	CG15884	Cpr97Eb	586	74	Lethal	Curled wing (See Fig. 6)
25455	CG1469	Ferritin 2 light chain	1307	76	Lethal	NP
23302	CG2216	Ferritin 1 heavy chain	1325	79	Lethal	NP
19100	CG15008	Cpr64Ac	573	80	NP	NP

RFABP, retinoid and fatty acid binding protein; NP, no phenotypic difference observed.

### RNAi of putative TG substrates

**Figure 6:**
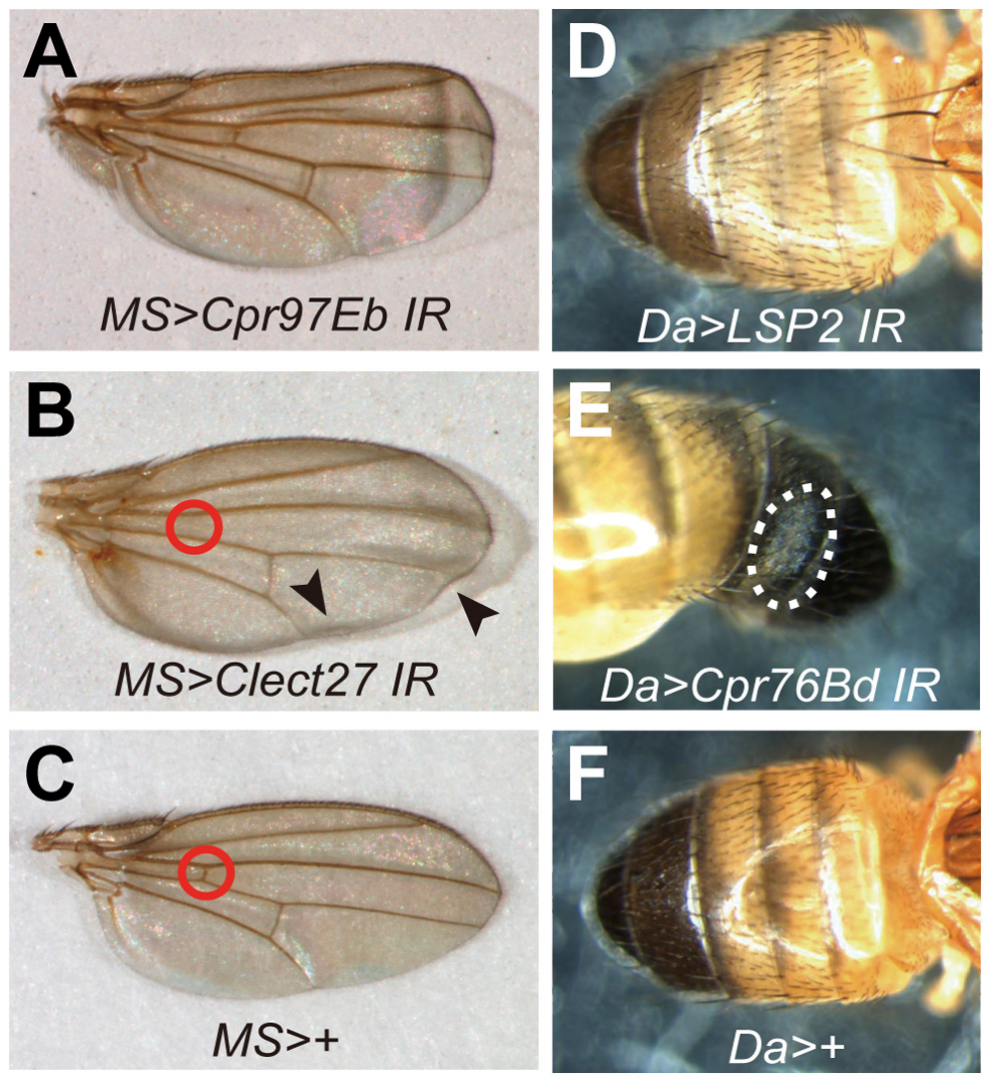
Phenotypes of TG substrate RNAi flies. Phenotypes of the *MS1096-GAL4>UAS-Cpr97Eb IR* (A), *MS1096-GAL4>UAS-Clect27 IR* (B), *Da-GAL4>UAS-LSP2 IR* (D) and *Da-GAL4>UAS-Cpr76Bd IR* (E) flies. The control flies, *MS-GAL4*>+ (C) and *Da-GAL4*>+ (F), are also indicated. Each fly was laid at 25°C. *MS, MS1096-GAL4; Da, Da-GAL4*.

We characterized the phenotypes of flies in which RNAi targeted each of these 12 genes in Table 1. Knocking down experiments of seven genes including *CG11064*, *CG6186*, *CG13214*, *CG3244, CG15884*, *CG1469*, and *CG2216* resulted in a lethal phenotype, and those of *CG9299* (*Cpr76Bd*) and *CG6806* (*larval serum protein 2, LSP2*) resulted in abnormal cuticle morphology. In contrast, those of the other three genes resulted in neither lethality nor phenotypic abnormality. Since the *Da-GAL4* driver promoted a lethal phenotype for the seven genes, we used an *MS1096-GAL4* driver, for which expression is restricted to the wing disc. The wing disc-restricted knocking down resulted in phenotypic abnormality for *CG15884* and *CG3244*, which respectively coded Cpr97Eb protein and a putative C-type lectin with a molecular mass of 27 kDa (tentatively designated Clect27). *MS1096-GAL4>UAS-Cpr97Eb IR* flies exhibited curled wings (Figure 6A), whereas, *MS1096-GAL4>UAS-Clect27 IR* flies had a wrinkled morphology (Figure 6B, arrowheads) and lacked the anterior crossvein (Figure 6B, a circle). Several melanized segments on the abdominal tergite in the adult flies were faded and variegated in *Da-GAL4>UAS-LSP2 IR* and *Da-GAL4>UAS-Cpr76Bd IR* flies, respectively (Figures 6D and 6E).

### Characterization of recombinant proteins of the putative TG substrates

**Figure 7:**
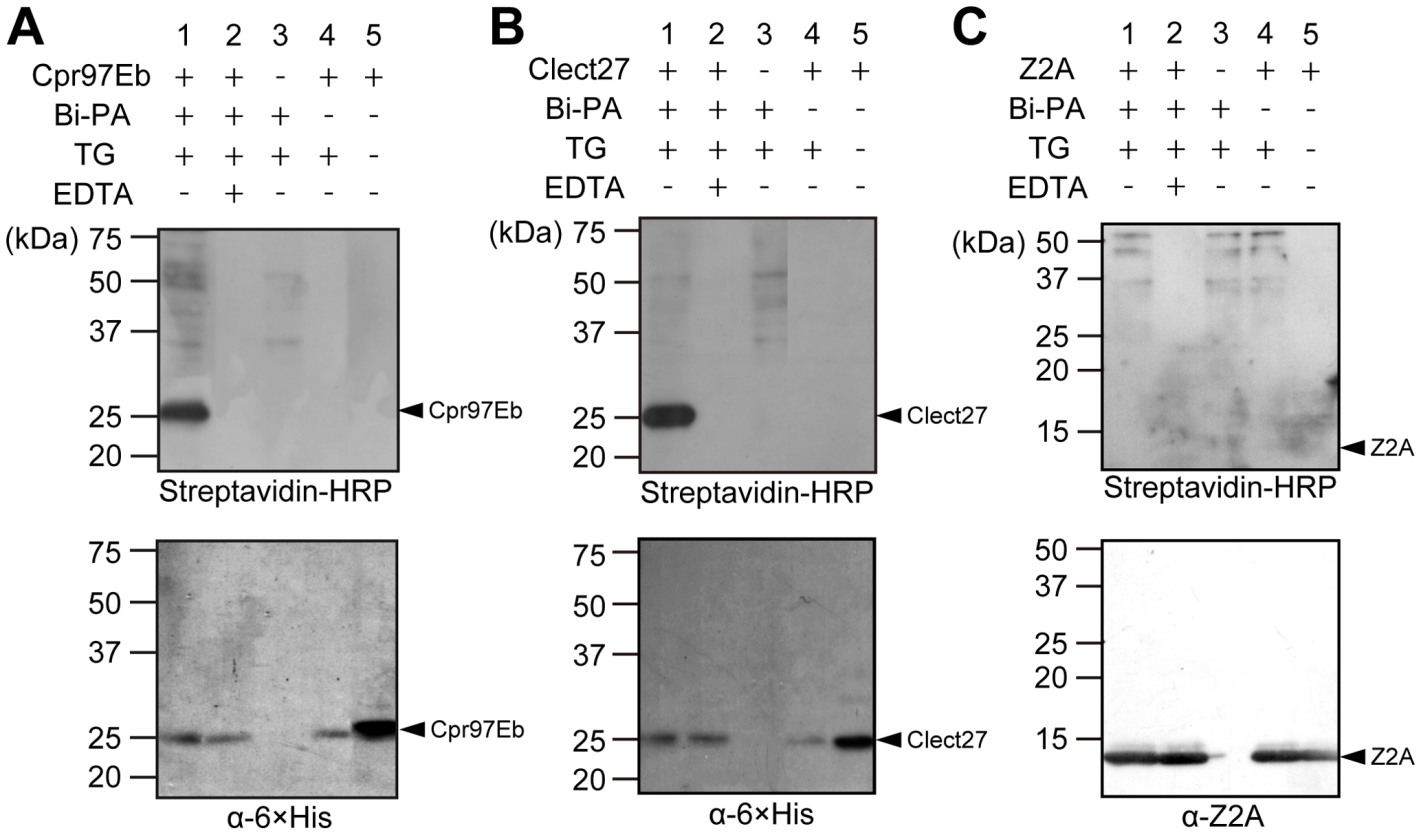
TG-dependent incorporation of Bi-PA to Cpr97Eb and Clect27 proteins. Cpr97Eb (A) and Clect27 (B) proteins were incubated with or without Bi-PA in the presence of TG. The incorporation of Bi-PA was detected with biotinylated streptavidin-HRP (upper panel). Z2A (C) is a recombinant version of horseshoe crab β-1,3-D-glucan-binding protein [43], which was used as negative control for the TG-dependent incorporation. Loaded recombinant proteins were detected by Western blotting with an anti-6×His tag antibody or anti-Z2A antibody (lower panels).

**Figure 8:**
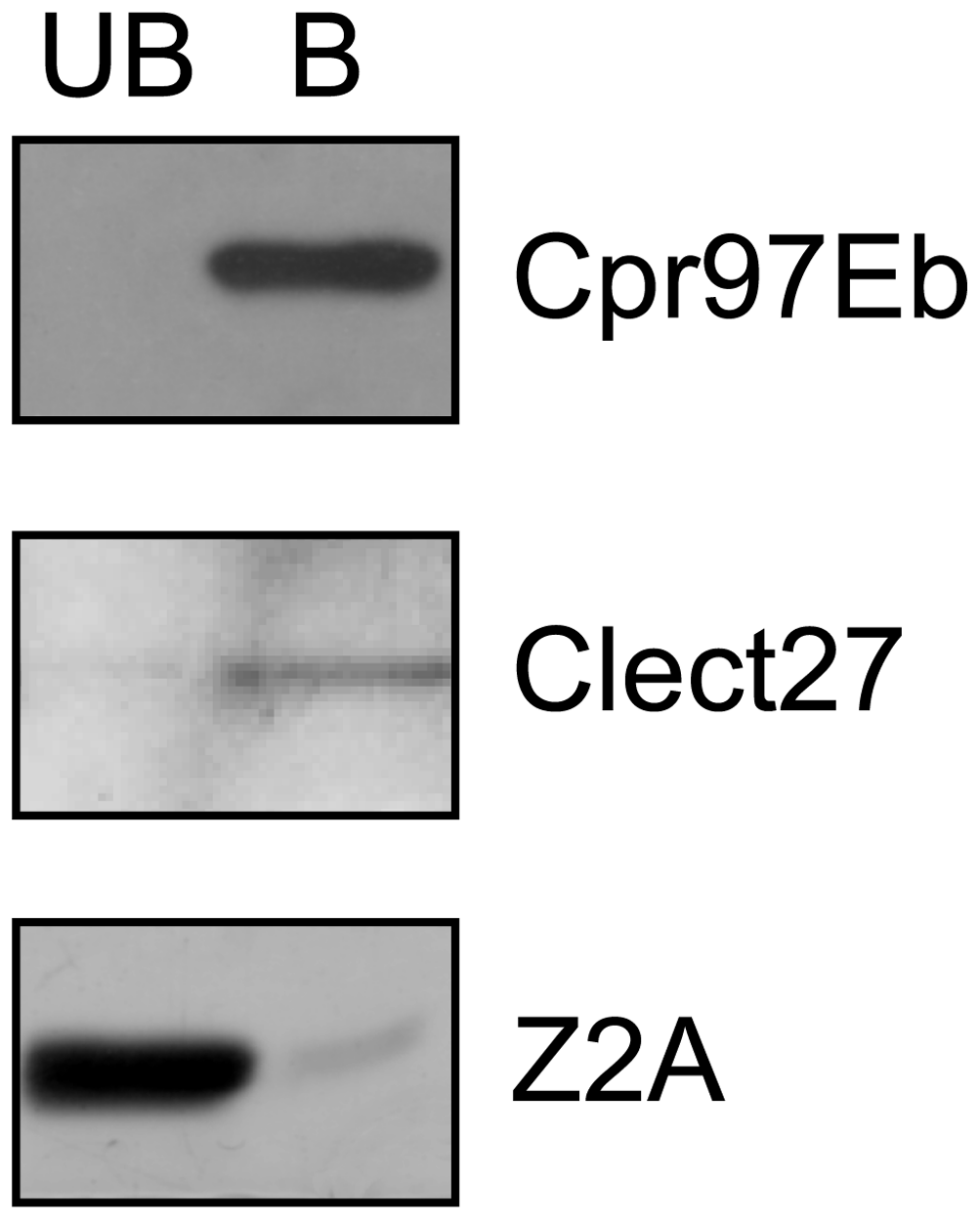
Binding of Cpr97Eb and Clect27 proteins to chitin. Cpr97Eb and Clect27 proteins were mixed with chitin, and unbound (UB) and bound (B) fractions were subjected to SDS-PAGE. The bound fraction was eluted by 2% SDS. Z2A was used as negative control for chitin binding. Proteins were detected by Coomassie Brilliant Blue R-250 staining (Clect27 and Z2A) or by anti-6×His tag antibody (Cpr97Eb).

In order to determine whether the putative TG substrates including Cpr76Bd, LSP2, Cpr97Eb, and Clect27 could serve as TG substrates, recombinant forms of these proteins were generated in *E. coli* with C-terminal His-tags. However, expression levels for Cpr76Bd and LSP2 in *E. coli* were too low to obtain their recombinant forms. After purification by nickel affinity and ion-exchange chromatography, Cpr97Eb and Clect27 were incubated with 5-biotinamidopentylamine (Bi-PA) in the presence of TG, subjected to SDS-PAGE, and analyzed by western blotting with biotinylated streptavidin-horseradish peroxidase (HRP). Incorporation of Bi-PA into Crp97Eb and Clect27 proteins was observed (lane 1 in Figures 7A and 7B), which was inhibited by EDTA, indicating that TG activity is dependent on the presence of divalent cations (lane 2 in Figures 7A and 7B). Bi-PA was also incorporated into several unknown proteins derived from TG fraction used (lane 3 in Figures 7A and 7B). Intermolecular reaction to form a homopolymer of Cpr97Eb or Clect27 protein by TG was not observed in the absence of Bi-PA, suggesting that Cpr97Eb and Clect27 proteins may undergo heterotypic TG-dependent crosslinking (lane 4 in Figures 7A and 7B). In addition, Clect27 and Cpr97Eb proteins exhibited binding activity to chitin (Figure 8).

### Measurements of transcuticular water loss of *TG*-RNAi flies

We hypothesized that cuticular abnormalities of *TG*-RNAi flies cause transcuticular water loss. To estimate water content, wet and dry weights of whole body of adult *TG*-RNAi flies at 3 and 15 days after eclosion were measured. The wet/dry ratios of *TG*-RNAi flies were respectively 3.30±0.72 and 3.17±0.26, which were consistent with those of the wild type flies (3.20±0.06 and 3.46±0.05, respectively), indicating that the shorter life span of *TG*-RNAi flies is not caused by transcuticular water loss: no significant difference between *TG*-RNAi files and the wild type flies was found by the *G*-test analysis (*G* = 8.13×10^−5^, d.f. = 1, *P* = 0.993 at 3 days after eclosion; *G* = 0.00278, d.f. = 1, *P* = 0.958 at 15 days after eclosion).

## Discussion

In this study, RNAi of the *TG* gene product using the ubiquitously expressed *Da-GAL4* driver caused a pupal semi-lethal phenotype and abnormal morphology at 25°C, (Figures 3 and 4), indicating that TG is important for *Drosophila* development and morphogenesis. Recently, Wang *et al*. reported that a *TG*-RNAi strain with reduced expression of TG using an *ACt5C-GAL4* driver showed no morphological defects at 22°C [16]. This discrepancy in the phenotypes for *TG*-RNAi flies of the two strains may be due to a more severe reduction level of TG in our RNAi flies by the different driver at higher temperature. The temperature dependence of *GAL4-UAS* expression system is widely accepted, and by altering the temperature, a wide range of expression levels of any responder can be achieved [25]. For example, to obtain information about the function of *Drosophila* POMT1 (protein *O*-mannosyltransferase-1), the *Act5C-GAL4/UAS-POMT-1-IR* fly was raised at 25°C and 28°C, and the fly showed a viability of 19% at 25°C, but 0% at 28°C, indicating that the knockdown is more effective at 28°C [26]. Interestingly, Wang *et al*. reported that the *TG*-knockdown flies increase mortality after entomopathogenic nematode infections, suggesting TG-dependent clot formation works as an important effector by helping to prevent septic infections [16]. Here we have demonstrated an increase of TG antigen and a concomitant increase in TG activity as a result of wounding, a finding that also suggests that TG is involved in the early phase of the innate immune reaction (Figure 2). *TG*-RNAi flies showed a significantly shorter life span than their counterparts (Figure 4A). *TG*-1 knockout mice die of severe dehydration due to high transepidermal water loss within 4–5 h after birth, caused by defective skin barrier development [27]. However, *TG*-RNAi flies did not appear to die of transcuticular water loss, suggesting that TG may have another pivotal function for survival.

We identified four putative TG substrates, namely Cpr97Eb, Cpr76Bd, LSP2, and CG3244 (here designated Clect27) by mass spectrometry and evaluated their functions by RNAi (Table 1). The *Da-GAL4* driver promoted lethal phenotypes for *Crp97Eb* and *Clect27* knockdowns, whereas the wing disc-restricted driver promoted abnormal wing morphologies (Figures 6A and 6B). The TG-dependent incorporation of Bi-PA into Crp97Eb and Clect27 proteins further implicated these proteins as potential TG substrates *in vivo* (Figure 7). Crp97Eb protein contains a Rebers and Riddiford consensus sequence, which is found in arthropod cuticular chitin-binding proteins [17], [18], [21], [28]. As expected, recombinant Cpr97Eb protein exhibited chitin-binding activity (Figure 8). *Cpr97Eb* gene is strongly expressed during pupal wing morphogenesis [29], and the morphological defect of the wing disc-restricted RNAi flies in this study suggests that Cpr97Eb protein is involved in the TG-dependent crosslinking required for cuticle morphogenesis and sclerotization. Clect27 protein is a putative galactose-binding C-type lectin based on the amino acid sequence with unknown physiological function. *Clect27* gene expression shows a highly localized pattern in the wing disc [30]. The wing disc-restricted *Clect27*-RNAi flies lacked anterior crossveins (Figure 6B, a circle) and recombinant Clect27 protein exhibited chitin-binding activity (Figure 8). Taken together, these findings suggest that Clect27 protein expressed in the wing may bind to the cuticle and be crosslinked by TG, and raise the possibility that a defect in this process may underlie the observed lack of the anterior crossvein in the wings of wing disc-restricted *Clect27*-RNAi flies.

RNAi of *Cpr76Bd* and *LSP2* resulted in faded or variegated black lines on the abdominal tergite, suggesting that these TG substrates are associated with melanin formation in the cuticle (Figures 6D and 6E). Although the physiological function of Cpr76Bd protein remains unknown, it contains Rebers and Riddiford consensus sequence, suggesting a cuticular chitin-binding activity. LSP2 is one of the major protein in hemolymph at 3L, and transcription of *LSP2* gene is controlled by 20-hydroxyecdysone [31], [32]. The LSP2 homo-hexamer is synthesized in the fat body and secreted into hemolymph [33], [34]. LSPs act mainly as storage proteins that provide energy and amino acids during metamorphosis [35], [36]. Injection of a larval serum protein, calliphorin from the blowfly *Calliphora vicina* labeled with [^14^C]phenylalanine, into larvae showed that the relatively high portion of calliphorin and/or calliphorin-derived phenylalanines are incorporated into SDS-insoluble portion of adult cuticle, suggesting the possible conversion of calliphorin into tanned insoluble proteins, and/or hydroxylation of phenylalanine to tyrosine involved in cuticular sclerotization [37]. Moreover, fractionation of sclerotized pupal cuticle showed that calliphorin forms covalent and non-covalent links with other cuticle components. Thus, LSP2 may be incorporated into cuticle by TG activity and involved in melanization, although the order of events in cuticle hardening due to TG and melanization remains to be clarified.

TG requires Ca^2+^ for activation [3]. Horseshoe crab TG is stored in hemocytes as a latent form under the low concentration of Ca^2+^ (∼1 µM) in cytosol and secreted in response to stimulation by lipopolysaccharides [10]. The secreted TG is activated immediately by Ca^2+^ in hemolymph plasma (∼10 mM), which is very important for the crosslinking of coagulin with TG substrates at injured sites to stop bleeding and to immobilize invading microbes [12], and the activated TG also catalyzes crosslinking of cuticular chitin-binding proteins secreted from the sub-cuticular epithelial cells [22]. Although in *Drosophila*, tissue localization of TG remains unknown, TG must be regulated timely and spatially by the Ca^2+^ concentration. Upon eclosion, the wings are expanded by blood pressure, a process that is completed within one hour [24]. TG secreted from cells in response to appropriate stimulation must be activated by Ca^2+^ in hemolymph plasma and transferred into the whole parts of the wings, and TG must crosslink several proteins to support wing maturation. Indeed, TG antigen existed in the wings of wild type flies immediately after eclosion (Figure 5, Wild type). In *TG*-RNAi flies, several proteins remained intact without crosslinking in the wings, resulting in wing blisters. This indicates that TG plays a critical role in the hardening steps during wing formation. During wing formation in *Drosophila*, an appropriate programmed cell death in epidermal cells is required prior to tanning and hardening [38]. In mammals, TG plays an important role in apoptosis to prevent the leakage of cytosolic components by protein crosslinking [39], [40]. *Drosophila* TG may be also involved in an apoptotic step in wing formation.

Mice lacking *TG-1* display a defective skin-barrier function and deficient wound repair [27]. A *Drosophila* transcription factor grainy head regulates enzymes, such as dopa decarboxylase and tyrosine hydroxylase, both of which catalyze the production of quinones, leading to covalent crosslinking between cuticle proteins and cuticular structural components [41]. Mice lacking *grainy head-like 3*, a homologue of *Drosophila grainy head*, display the same defects as in the case of the *TG*-1 knockout mice, accompanied by reduced expression of *TG-1* [42]. The epidermis-barrier function and the wound-repair pathway seem to be evolutionarily well conserved between mammals and *Drosophila*, suggesting that *Drosophila* could serve as a sophisticated model system to elucidate the molecular mechanisms underlying mammalian skin disorders.

## Materials and Methods

### Fly stocks

Flies were maintained on the standard *Drosophila* medium at 18, 25 or 29°C. Flies, *white* (*w^1118^*), *Da-GAL4* and *Tub-GAL80^ts^; Da-GAL4* were obtained from the Bloomington Stock Center. *MS1096-GAL4* strain was a gift from Dr. Ulrich Theopold at Stockholm University. *UAS-TG IR*, *UAS-Cpr76Bd IR*, *UAS-Clect27 IR*, and *UAS-LSP2 IR* flies were obtained from Dr. Ryu Ueda at the National Institute of Genetics, Mishima, Japan. *UAS-Cpr97Eb IR* flies were obtained from the Vienna *Drosophila* RNAi Center. *UAS-TG* strain was gift from Drs. Koji Ikura and Akira Ichikawa. Strain *w^1118^* was used as the wild type.

### Preparation of polyclonal antibodies against TG

To prepare polyclonal antibodies, the full-length, the N-terminal region (residues 1–284), and the C-terminal region (515–776) of TG were expressed in *E. coli* strain BL21 (DE3) pLysS (Novagen). An expression level of the full-length of TG was too low to obtain the recombinant form as an antigen. Inclusion bodies containing the recombinant proteins of the N-terminal region and the C-terminal region of TG were isolated, subjected to SDS-PAGE under reducing conditions, and stained using negative staining. The protein bands corresponding to the recombinant proteins were excised from the gel and recovered by electroelution for the immunization of rabbits (Asahi Techno Glass, Chiba, Japan). An antibody titer of the anti-serum against the C-terminal region of TG did not rise after several boosts of the antigen. Therefore, an polyclonal antibody against the N-terminal region of TG was purified sequentially from the anti-serum by using protein A-Sepharose and antigen-conjugated Affi-Gel-10 (Bio-Rad Laboratories, Hercules, CA). The resulting antibody cross-reacted with horseshoe crab TG in hemocyte lysates by Western blotting.

### Extraction of proteins from the whole body and wings

Flies were homogenized in 1% Nonidet P-40 in 50 mM Tris-acetate, pH 7.5, containing 150 mM NaCl, 1 mM EDTA and 1 mM phenylmethylsulfonyl fluoride, and centrifuged at 20,000× g at 4°C for 15 min to collect the supernatant. For wing protein extraction, wings were collected, washed with 70% ethanol, and homogenized in 10% acetic acid using a pellet mixer. After incubation at 4°C for 16 h, the homogenate was centrifuged at 20,000× g at 4°C for 15 min, after which the supernatant was lyophilized.

### Western blotting

Samples were subjected to SDS-PAGE and transferred to PVDF membrane. After blocking with 5% dry milk, the membrane was incubated with the anti-TG antibody or anti-6×His tag antibody (Nacalai Tesque, Kyoto, Japan) and then with the secondary antibody (horseradish peroxidase-conjugated (HRP) goat anti-rabbit or -mouse IgG, Bio-Rad Laboratories), followed by development with Chemi-Lumi One (Nacalai Tesque). β-Tubulin was detected with a mouse anti-tubulin antibody (Chemicon International, Temecula, CA).

### Enzyme-linked immunosorbent assay

Microtiter plates were coated with homogenates of 10 individuals (whole body) at 37°C for 1 h. After washing with 50 mM Tris-HCl, pH 7.5, containing 150 mM NaCl, wells were blocked with 5% dry milk in the same buffer. Plates were incubated with the anti-TG antibody at 37°C for 1 h and then with 5,000-fold diluted goat anti-rabbit IgG-HRP conjugate (Bio-Rad Laboratories), and developed using o-phenylenediamine substrate with detection at 490 nm.

### TG activity assays

Microtiter plates were coated with 50 µl of *N, N'*-dimethylcasein (15 mg/ml; Sigma, St. Louis, MO) at 4°C overnight, and the wells were subsequently blocked with 0.5% dry milk in 0.1 M Tris-HCl, pH 8.5 at 37°C for 1 h and washed with 0.1 M Tris-HCl, pH 8.5. Reagents were added to each well as follows: 10 mM CaCl_2_, 10 mM dithiothreitol, 0.5 mM 5-biotinamidopentylamine (Bi-PA; Pierce Chemical, Rockford, IL), whole body lysate (2.5 mg protein), and 0.1 M Tris-HCl, pH 8.5 (total volume of 50 µl per well). The microtiter plates were incubated at 37°C for 1 h, and the reaction was stopped by washing with 200 mM EDTA followed by washing with 0.1 M Tris-HCl, pH 8.5. The biotinylated streptavidin-HRP conjugate (GE Healthcare, Buckinghamshire, UK) diluted 1∶200 with 0.5% dry milk in 0.1 M Tris-HCl, pH 8.5 was added to each well and incubated at 37°C for 1 h. The plate was washed once with 0.001% Triton X-100 followed by four washes with 0.1 M Tris-HCl, pH 8.5. The activity of horseradish peroxidase was detected with o-phenylenediamine at 490 nm.

TG activity was additionally assayed by fluorometric measurement of monodansylcadaverine incorporation into *N*, *N'*-dimethylcasein. Homogenates of eight individual flies were incubated with 50 mM Tris-acetate, pH 7.5, containing 10 mM CaCl_2_, 10 mM dithiothreitol, 0.5 mM monodansylcadaverine, and 0.04% of *N*, *N'*-dimethylcasein at 37°C for 30 min, after which the reaction was stopped by adding 10% trichloroacetic acid. The resulting precipitate was solublized with 50 mM Tris-acetate, pH 7.5, containing 8 M Urea and 0.5% SDS. The amount of the incorporated monodansylcadaverine was quantitated with a fluorescence spectrophotometer.

### Statistical analyses

TG activity or the amount of TG antigen was analyzed by the ANOVA. The model of the ANOVA was as follows: *Y_ij_*  =  *u* + *A_i_* + *e_ij_*, where *Y* is the TG activity, *u* is the overall mean, *A_i_* is the *i*th developmental stage effect or injury time effect and *e_ij_* is the error term. The developmental stage effect or injury time effect was considered as the fixed effect. Bonferroni correction for multiple comparisons was applied to evaluate the pairwise difference in average activity or average amount of TG antigen between developmental stages or injury times. The log-rank test was performed to compare survival in two groups (*TG*-RNAi and *Da-GAL4>+* flies). Differences of transcuticular water loss between wild type and *TG*-RNAi flies were tested using the *G* test for goodness of fit.

### Mass spectrometry

Proteins were subjected to SDS-PAGE and stained with Coomassie Brilliant Blue R-250. Protein bands were excised, digested with trypsin, and subjected to LC/MS/MS analysis. Peak lists obtained from the mass spectra were used to identify proteins using the Mascot search engine (Matrixscience).

### Expression of recombinant Cpr97Eb and Clect27 in *E. coli*

To construct expression vectors, cDNA fragments were amplified by PCR. An amplimer encoding the entire *Clect27* coding sequence were inserted into expression vector pET-22b (Novagen) between the NdeI and XhoI sites. An amplimer the entire *Cpr97Eb* coding sequence was inserted into pET-22b between the NcoI and XhoI sites. All constructs were verified by DNA sequencing. These constructs, which contain C-terminal His-tags, were expressed in the *E. coli* strain Origami B (DE3) (Novagen). Bacteria were cultured in Luria-Bertani medium, and expression was induced by the addition of isopropyl-β-D-thiogalactoside at a final concentration of 30 µM at 18°C for 20 h. Bacterial pellets were harvested by centrifugation and sonicated in 20 ml of 50 mM Tris-HCl, pH 8.2, containing 150 mM NaCl, 1% Nonidet P-40 and 1 mM phenylmethylsulfonyl fluoride. After sonication, supernatants were recovered by centrifugation and purified according to the manufacturer's protocol using Ni-NTA agarose. Eluates of Clect27 protein from Ni-NTA agarose were diluted with 50 mM Tris-HCl, pH 7.0, containing 100 mM NaCl and further purified on a DEAE Sepharose CL-6B column (1×2 cm). Proteins were eluted with a linear NaCl gradient (100–500 mM) in the same buffer.

### Incorporation of Bi-PA into Clect27 and Cpr97Eb proteins

Recombinant proteins were incubated with TG in 50 mM Tris-HCl, pH 8.5, containing 10 mM CaCl_2_, 10 mM dithiothreitol and 1 mM Bi-PA at 37°C for 1 h. Whole body extract of *Da-GAL4>UAS-TG* was used as the TG source. Following the reaction, aliquots were subjected to SDS-PAGE and electroblotted on PVDF membrane. After blocking with 5% dry milk, the membrane was incubated at room temperature for 1 h with the biotinylated streptavidin-HRP conjugate diluted 1∶1000 with 20 mM Tris-HCl, pH 7.5 containing 5% nonfat dry milk and 150 mM NaCl, followed by development with Chemi-Lumi One reagent.

### Binding assay for chitin

Chitin-binding assays were performed as previously reported [43]. Briefly, proteins were mixed with chitin in 50 mM Tris-HCl, pH 7.5, 150 mM NaCl, and incubated at 4°C for 10 min. Supernatants were separated by centrifugation and precipitates were washed with the same buffer. Protein bound to chitin was eluted with 2% SDS. Bound and unbound fractions were subjected to SDS-PAGE. Clect27 and Cpr97Eb were detected by staining with Coomassie Brilliant Blue R-250 and by Western blotting with an anti-6×His tag antibody, respectively.

### Measurements of wet and dry weights of *TG*-RNAi flies

Twenty adult *TG*-RNAi or wild-type flies were anesthetized and weighed (wet weights). Flies were then dried with a centrifugal vacuum concentrator (model 78120KT, Labconco, Kansas city, MO) for 6 h and weighed (dry weights).

### Optical and Scanning Electron Microscopies

Optical microscopic observation was performed with a Nikon SMZ 1000 microscope. For scanning electron microscopy, non-fixed samples without coating were directly observed by a Keyence VE-9800 scanning electron microscope.

## References

[1] Furie B, Furie BC (1988). The molecular basis of blood coagulation.. Cell.

[2] Kalinin A, Marekov LN, Steinert PM (2001). Assembly of the epidermal cornified cell envelope.. J Cell Sci.

[3] Lorand L, Graham RM (2003). Transglutaminases: crosslinking enzymes with pleiotropic functions.. Nat Rev Mol Cell Biol.

[4] Lorand L (2001). Factor XIII: structure, activation, and interactions with fibrinogen and fibrin.. Ann N Y Acad Sci.

[5] Kopacek P, Hall M, Söderhäll K (1993). Characterization of a clotting protein, isolated from plasma of the freshwater crayfish *Pacifastacus leniusculus*.. Eur J Biochem.

[6] Wang R, Liang Z, Hal M, Söderhäll K (2001). A transglutaminase involved in the coagulation system of the freshwater crayfish, *Pacifastacus leniusculus*. Tissue localisation and cDNA cloning.. Fish Shellfish Immunol.

[7] Theopold U, Schmidt O, Söderhäll K, Dushay MS (2004). Coagulation in arthropods: defense, wound closure and healing.. Trends Immunol.

[8] Karlsson C, Korayem AM, Scherfer C, Loseva O, Dushay MS (2004). Proteomic analysis of the *Drosophila* larval hemolymph clot.. J Biol Chem.

[9] Scherfer C, Karlsson C, Loseva O, Bidla G, Goto A (2004). Isolation and characterization of hemolymph clotting factors in *Drosophila melanogaster* by a pullout method.. Curr Biol.

[10] Osaki T, Kawabata S (2004). Structure and function of coagulogen, a clottable protein in horseshoe crab.. Cell Mol Life Sci.

[11] Kawasaki H, Nose T, Muta T, Iwanaga S, Shimohigashi Y (2000). Head-to-tail polymerization of coagulin, a clottable protein of horseshoe crab.. J Biol Chem.

[12] Osaki T, Okino N, Tokunaga F, Iwanaga S, Kawabata S (2002). Proline-rich cell surface antigens of horseshoe crab hemocytes are substrates for protein-crosslinking with a clotting protein coagulin.. J Biol Chem.

[13] Matsuda Y, Osaki T, Hashii T, Koshiba T, Kawabata S (2007). A cysteine-rich protein from an arthropod stabilizes clotting mesh and immobilizes bacteria at injury site.. J Biol Chem.

[14] Lustigman S, Brotman B, Huima T, Castelhano AL, Singh RN (1995). Transglutaminase-catalyzed reaction is important for molting of *Onchocerca volvulus* third-stage larvae.. Antimicrob Agents Chemother.

[15] Lin X, Söderhäll K, Söderhäll I (2008). Transglutaminase activity in the hematopoietic tissue of a crustacean, *Pacifastacus leniusculus*, importance in hemocyte homeostasis.. BMC Immunol.

[16] Wang Z, Wilhelmsson C, Hyrsl P, Loof TG, Dobes P (2010). Pathogen entrapment by transglutaminase—a conserved early innate immune mechanism.. PLoS Pathogens.

[17] Willis JH (1999). Cuticular proteins in insects and crustaceans.. Am Zool.

[18] Andersen SO, Peter MG, Roepstorff P (1996). Cuticular sclerotization in insects.. Comp Biochem Physiol.

[19] Sugumaran M (1998). Unified mechanism for sclerotization of insect cuticle.. Adv Insect Physiol.

[20] Kramer KJ, Kanost MR, Hopkins TL, Jiang H, Zhu YC (2001). Oxidative conjugation of catechols with proteins in insect skeletal systems.. Tetrahedron.

[21] Iijima M, Hashimoto T, Matsuda Y, Nagai T, Yamano Y (2005). Comprehensive sequence analysis of horseshoe crab cuticular proteins and their involvement in transglutaminase-dependent crosslinking.. FEBS J.

[22] Matsuda Y, Koshiba T, Osaki T, Suyama H, Arisaka F (2007). An arthropod cuticular chitin-binding protein endows injured sites with transglutaminase-dependent mesh.. J Biol Chem.

[23] Baker JD, Truman JW (2002). Mutation in the *Drosophila* glycoprotein hormone receptor, rickets, eliminate neuropeptide-induced tanning and selectively block a stereotyped behavioral program.. J Exp Biol.

[24] Kiger JA, Natzle JE, Kimbrell DA, Paddy MR, Kleinhesselink K (2007). Tissue remodeling during maturation of the *Drosophila* wing.. Dev Biol.

[25] Duffy JB (2002). GAL4 system in *Drosophila*: a fly geneticist's Swiss army knife.. Genesis.

[26] Ichimiya T, Manya H, Ohmae Y, Yoshida H, Takahashi K (2004). The twisted abdomen phenotype of *Drosophila* POMT1 and POMT2 mutants coincides with their heterophilic protein O-mannosyltransferase activity.. J Biol Chem.

[27] Matsuki M, Yamashita F, Ishida-Yamamoto A, Yamada K, Kinoshita C (1998). Defective stratum corneum and early neonatal death in mice lacking the gene for transglutaminase 1 (keratinocyte TG).. Proc Natl Acad Sci USA.

[28] Karouzou MV, Spyropoulos Y, Iconomidou VA, Cornman RS, Hamodrakas SJ (2007). *Drosophila* cuticular proteins with the R&R Consensus: Annotation and classification with a new tool for discriminating RR-1 and RR-2 sequences.. Insect Biochem Mol Biol.

[29] Ren N, Zhu C, Lee H, Adler PN (2005). Gene Expression During *Drosophila* Wing Morphogenesis and Differentiation.. Genetics.

[30] Butler JM, Jacobsen LT, Cain MD, Jarman GM, Hubank M (2003). Discovery of genes with highly restricted expression patterns in the *Drosophila* wing disc using DNA oligonucleotide microarrays.. Development.

[31] Roberts DB, Wolfe J, Akam ME (1977). The developmental profiles of two major hemolymph proteins from *Drosophila melanogaster*.. J Insect Physiol.

[32] Mousseron-Grall S, Kejzlarova-Lepesant J, Burmester T, Chihara C, Barray M (1997). Sequence, structure and evolution of the ecdysone-inducible *Lsp-2* gene of *Drosophila melanogaster*.. Eur J Biochem.

[33] Akam ME, Roberts DB, Richards GP, Ashburner M (1978). *Drosophila*: the genetics of two major larval proteins.. Cell.

[34] Akam ME, Roberts DB, Wolfe J (1978). *Drosophila* hemolymph proteins: purification, characterization, and genetic mapping of larval serum protein 2 in *D. melanogaster*.. Biochem Genet.

[35] Levenbook L, Bauer AC (1984). The fate of the larval storage protein calliphorin during adult development of *Calliphora vicina*.. Insect Biochem.

[36] Telfer WH, Kunkel JG (1991). The function and evolution of insect storage hexamers.. Annu Rev Entomol.

[37] König M, Agrawal OP, Schenkel H, Scheller K (1986). Incorporation of calliphorin into the cuticle of the developing blowfly, *Calliphora vicina*.. Roux's Arch Dev Biol.

[38] Kimura K, Kodama A, Hayasaka Y, Ohta T (2004). Activation of the cAMP/PKA signaling pathway is required for post-ecdysial cell death in wing epidermal cells of *Drosophila melanogaster*.. Development.

[39] Fesus L, Thomazy V, Falus A (1987). Induction and activation of tissue transglutaminase during programmed cell death.. FEBS Lett.

[40] Melino G, Annicchiarico-Petruzzelli M, Piredda L, Candi E, Gentile V (1994). Tissue transglutaminase and apoptosis: sense and antisense transfection studies with human neuroblastoma cells.. Mol Cell Biol.

[41] Mace KA, Pearson JC, McGinnis W (2005). An epidermal barrier wound repair pathway in *Drosophila* is mediated by *grainy head*.. Science.

[42] Ting SB, Caddy J, Hislop N, Wilanowski T, Auden A (2005). A homolog of *Drosophila grainy head* is essential for epidermal integrity in mice.. Science.

[43] Ueda Y, Ohwada S, Abe Y, Shibata T, Iijima M (2009). Factor G utilizes a carbohydrate-binding cleft that is conserved between horseshoe crab and bacteria for the recognition of β-1,3-D-glucans.. J Immunol.

